# Assessment of the knowledge level and experience of healthcare personnel concerning CPR and early defibrillation: an internal survey

**DOI:** 10.1186/s12872-021-02009-2

**Published:** 2021-04-20

**Authors:** G. Spinelli, E. Brogi, A. Sidoti, N. Pagnucci, F. Forfori

**Affiliations:** Department of Anesthesia and Intensive Care, University of Pisa, Via Paradisa 2, 56124 Pisa, Italy

**Keywords:** Heart arrest, Cardiopulmonary resuscitation, Advanced cardiac life support, Professional education

## Abstract

**Background:**

In‐hospital cardiac arrest (IHCA) is a major public health problem with significant mortality. Rapid cardiopulmonary resuscitation and early defibrillation is extremely connected to patient outcome. In this study, we aimed to assess the effects of a basic life support and defibrillation course in improving knowledge in IHCA management.

**Methods:**

We performed a prospective observational study recruiting healthcare personnel working at Azienda Ospedaliero Universitaria Pisana, Pisa, Italy. Study consisted in the administration of two questionnaires before and after BLS-D course. The course was structured as an informative meeting and it was held according to European Resuscitation Council guidelines.

**Results:**

78 participants completed pre- and post-course questionnaires. Only 31.9% of the participants had taken part in a BLS-D before our study. After the course, we found a significative increase in the percentage of participants that evaluated their skills adequate in IHCA management (17.9% vs 42.3%; *p* < 0.01) and in the correct use of defibrillator (38.8% vs 67.9% *p* < 0.001). However, 51.3% of respondents still consider their preparation not entirely appropriate after the course. Even more, we observed a significant increase in the number of corrected responses after the course, especially about sequence performed in case of absent vital sign, CPR maneuvers and use of defibrillator.

**Conclusions:**

The training course resulted in significant increase in the level of knowledge about the general management of IHCA in hospital staff. Therefore, a simple intervention such as an informative meetings improved significantly the knowledge about IHCA and, consequently, can lead to a reduction of morbidity and mortality.

**Supplementary Information:**

The online version contains supplementary material available at 10.1186/s12872-021-02009-2.

## Background

In‐hospital cardiac arrest (IHCA) represents a major public health issue with significant mortality [1]. After a review of the published literature, the survival to discharge rate of IHCA varies widely (i.e., from 0 to 42%) with an incidence of 1–5 cases each 1000 patients [2–6]. Ventricular fibrillation in adults and pulseless electrical activity or asystole in pediatrics are the more common presenting rhythm [7]. Due to the different pathophysiological mechanisms (i.e., coronary artery disease in adults vs hypoxia in pediatric patients), cardiopulmonary resuscitation protocols differ between these two groups of patients [8].

The chain of survival refers to a series of time-sensitive actions with the aim of improving survival rate; each critical intervention is tightly linked to the next [9]. Consequently, all healthcare personnel should know “when” and “how” to perform the various steps correctly and promptly. The recognition of the clinical signs of cardiac arrest (i.e., loss of consciousness, abnormal breathing patterns and no pulse) is linked to the call of the Rapid Response Team (RRT). Then, early cardiopulmonary resuscitation (high-quality CPR and early defibrillation) represents the subsequent step. However, in order to provide the best level of care, education (e.g., simulation, “low dose high frequency” training) and the implementation of resources (e.g., dispatcher assisted CPR, case reviews, feedback, high fidelity manikins) represent further key factors that have to be taken into account. The Utstein Formula of Survival was described for the first time in 2003 by the International Liaison Committee on Resuscitation (ILCOR) [10]. The formula had the aim of predicting survival rate from sudden cardiac arrest [11]. The three multiplicands are guidelines quality, efficient education of healthcare personal and local implementation. Consequently, the knowledge and the experience of healthcare personnel on cardiopulmonary resuscitation protocol and regular CPR training are considered vital factors with a huge impact on patient’s survival [12, 13]. Unfortunately, there is a gap between expectation and real skill retention leading to a weak response to emergencies specifically outside the critical area [14].

In the present study, we conducted an internal survey with the objective to analyze the level of education of the healthcare personal working in our hospital in the management of patients with cardiac arrest. We administered anonymous questionnaires before and after an educational course on BLS-D in order to assess training needs, basic knowledge and skills reached after the course.

## Methods

An internal survey was conducted in order to evaluate the general knowledge and skills of healthcare personnel working in our hospital. After approval of the Local Research Ethics Committee of Pisa a prospective observational study was conducted in a one-year period. We organized an “information meeting” on BLS-D and we enrolled nurses and medical staff working at the Pisa University Hospital-AOUP (Azienda Ospedaliero Universitaria Pisana). Our hospital is a tertiary referral hospital. The participants were doctor of medicine and qualified nurses both from critical and non-critical areas*.* Written informed consent was obtained from all the participants in the study. Data were gathered by the evaluation of the two questionnaires.

### Study protocol

The design of the study consisted of three-step phases:

1. Pre-course: Administration of a pre-course questionnaire to participants in order to evaluate their personal experience in cardiac arrest management (defined Questionnaire “A” as shown in Additional file 1: Material 1); then administration of a second questionary in order to evaluate their basic knowledge in ERC BLS-D guideline (defined Questionnaire “B” as shown in Additional file 1: Material 2);
2. Theoretical and practical BLS-D course according to the European Resuscitation Council (ERC) guidelines 2015[15];
3. Post-course: Administration of questionnaire regarding ERC BLS-D guideline to participants in order to verify the improvement in knowledge and skills in the management of IHCA (Questionnaire “B” Additional file 1: Material 2). Even more, during post-course phase, we asked to the participants to reply to question 11 (Are you able to use a defibrillator?) and question 18 (How can you judge your preparation on cardiac arrest management?) of Questionnaire “A”.
  The theoretical and practical training sessions were held by a certified instructor for BLS-D of the Italian Red Cross (S.G.) with experience in resuscitation and defibrillation teaching. The 2-h theoretical meeting were conducted in an interactive way and the topics covered were as follows:
  1. Common causes of in-hospital cardiac arrest;
  2. Prevention strategies;
  3. Objective of the BLS-D (in accordance with the ERC Guidelines 2015, the algorithm for in-hospital cardiac arrest treatment [15]).

CPR manikin for ALS (Advance Life Support), equipped with basic airway tools (i.e., self-inflating bag, oropharyngeal cannula, face mask), AED trainer and manual defibrillator trainer were used for demonstration purpose during the practical part. All participants were allowed to perform the BLS-D sequence on the training manikin under supervision and guidance. Participants were divided into groups of 5. Each group has spent more than 1 h of training.

Data were extracted from the two questionnaires. Both questionnaires were filled by the participants included in the final analysis. The response to the questionnaires and the attendance at the course were optional. The compilation of the initial questionnaire did not include mandatory attendance at the formative course, whereas all those who had taken part in the formative course must have completed the initial questionnaire.

### Questionnaires

Questionnaires administered to participants before and after the course were:

1. *Questionnaire regarding personal experience in cardiac arrest management (defined Questionnaire “A”)* 19 questions concerning theoretical knowledge and direct experience regarding cardiopulmonary resuscitation and early defibrillation (e.g., CPR and use of automatic defibrillator). Pre-course phase.
2. *ERC BLS-D guidelines (defined Questionnaire “B”)* 14 multiple-choice questions on cardiopulmonary resuscitation and early defibrillation according to the ERC Guidelines 2015. This questionnaire (showed in Additional file 1: Material 2) was administrated both during Pre-course step and *Post course* questionnaire phases.

A case report form was filled out with the abovementioned data for each participant involved in an anonymous form, data from questionnaire were collected before and after BLS-D course and compared.

### Statistical approach

The data were entered into a spreadsheet (Microsoft Excel) and analysed using SPSS (IBM SPSS software version 21). Data were divided in two groups according to the pre course or post-course phases. Data were shown as percentage. Mc Nemar test was performed for comparing pre-training and post-training group. A value of P below 0.05 defined the significance.

## Results

**Fig. 1:**
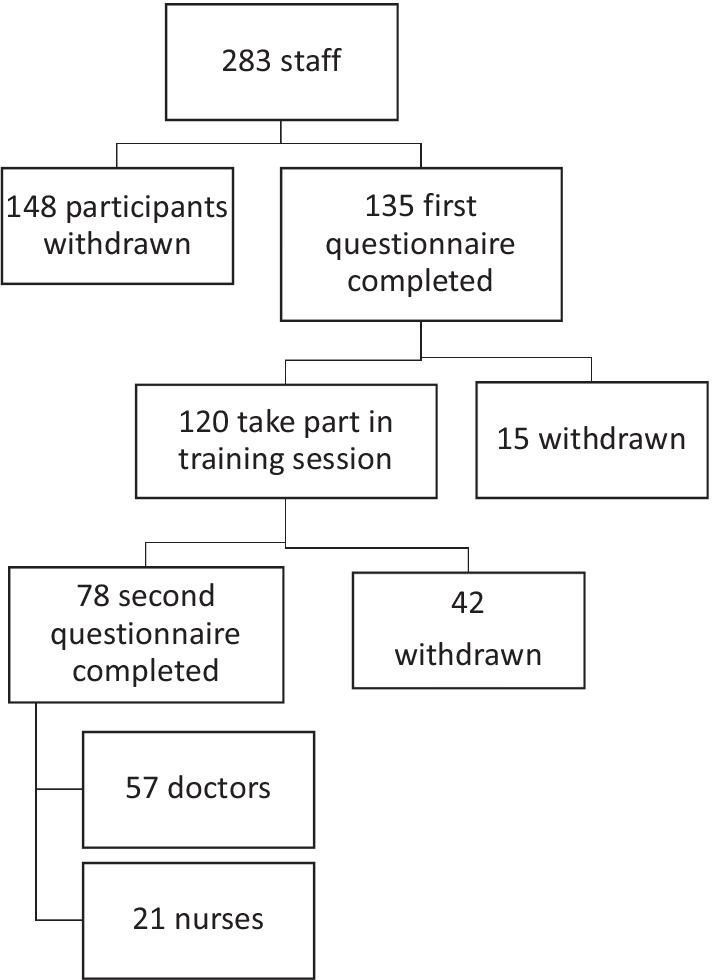
Flowchart of the participants included

The pre-course questionnaires were completed by 135 operators (97 doctors and 38 nurses), 120 participants followed the theoretical and practical training sessions (88.9%). The post course questionnaire was completed by 78 operators (57 doctors and 21 nurses, 57.8% of the total, and respectively 58.8% and 55.3%). A flowchart of the participants included is shown in Fig. 1. The participants were both from critical and non-critical areas (as shown in Table 1).

**Table 1:** Departments of the participants that completed the first questionnaire

Department	Number of participants	Percentage
Intensive Care Unit	25	18.5
Endocrine Surgery Unit	15	11.1
Day surgery Unit	10	7.4
Colorectal surgery Unit	11	8.1
General Medicine Unit	9	6.75
Nephrology Unit	20	14.8
Dialysis Unit	19	14.1
Otorhinolaryngology Unit	20	14.8
Urology	6	4.4
Total	135	100

**Fig. 2:**
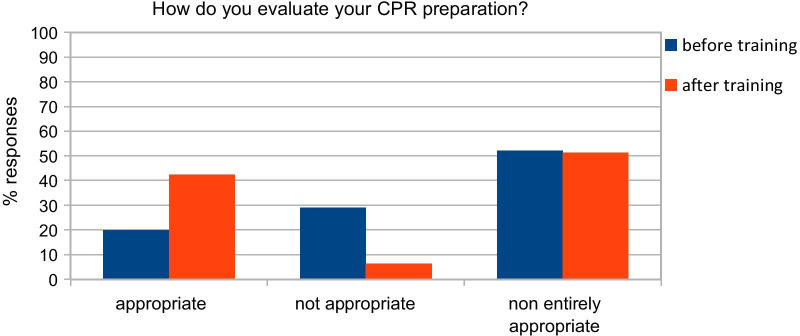
Subjective evaluation of preparation in cardiac arrest management: comparison between pre and post-course

**Fig. 3:**
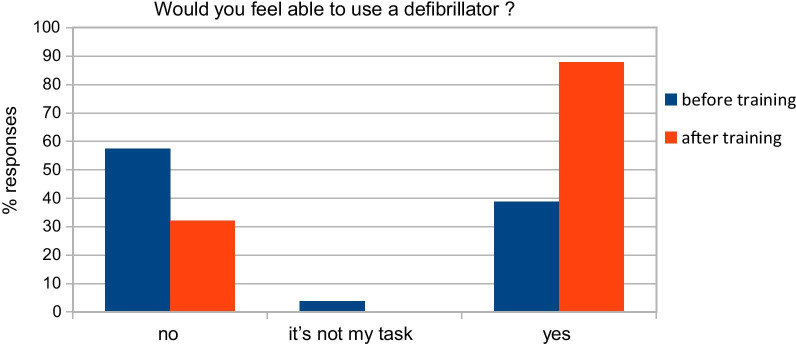
Subjective evaluation of preparation in using defibrillator: comparison between pre and post-course

Analyzing data from the pre-course questionnaire “A”, we observed that only 31,9% of the participants had taken part in a BLS-D, whereas 54.8% of them had taken part in BLS course. Therefore, 14% of the participants had never taken part in a BLS/BLS-D course. We also found that 73.9% had witnessed IHCA at least once; in 59% of the cases in the ward and 41% in the OR. In 46.5% of the cases, the patient had a return of spontaneous circulation, whereas in 34.3% of the cases the patient died (in the remaining 19.2% of the cases, participants did not know the outcome). Most of the participants (33.7%) performed resuscitation maneuvers by performing chest compressions and/or pulmonary ventilation. 15.3% did not take part in the resuscitation; the majority (79%) of participants stated that they did not take part in the maneuvers because others were already resuscitating the patient, 18.4% did not know what to do in that circumstance and 2.5% considered more appropriate to wait for the arrival of the emergency team before performing CPR. Only 17.9% of the staff considered adequate their skills, 29.9% not adequate and 52.2% not entirely adequate. After our training sessions, the percentage appreciably changed with 42.3% of the participants evaluated their preparation adequate. Remarkably, after training sessions, the percentage of participants that felt to retain inadequate skills appreciably reduced from 29.9 to 6.4%. 51.3% of respondents still consider their preparation not entirely appropriate (as shown in Fig. 2). Comparing pre-training and post-training groups with Mc Nemar test, we observed that the percentage of those who consider themselves well prepared was statistically significantly higher in post-training compared to pre-training questionnaire (*p* < 0.01). Before the training session, 97% were aware of the presence of a defibrillator in the ward but only 38.8% declared to be able to use it. After our training sessions 67.9% declared to be confident to use a defibrillator: with a statically significant difference after the practice session (*p* < 0.01, McNemar test, Fig. 3).

Analyzing data from the questionnaire “B”: Comparisons between the percentage of correct answers between pre- and post-training course were shown in Table 2. We observed an increase in the percentage of the correct response from pre-course and post course for every question (from 1 to 14). In particular, from question 1 to 7 the average of correct answers was 70.6% before and 96.4% after the training session. For questions 8–14, regarding the use of an AED defibrillator, the average of correct answers was 42.2% before and up to 89.50% after the course. Even more, it is important to highlight the significant increase in the percentage of correct response between pre-course and post-curse for the question number 6 (from 50.4 to 98.7%), question number 7 (from 23.6 to 94.9%), question number 9 (from 32 to 85.1%), question number 12 (from 19.3 to 93.6%) and question number 13 (from 51 to 88.5%).

**Table 2:** Percentage of corrected answers before and after training session to post course questionnaire-*ERC BLSD guideline knowledge*

Question	Pre training	Post training
1. Occurrence of neurons permanent damage consequently to cardiac arrest	89.9% N = 70	100% N = 78
2. Correct actions to do in unconscious patient, if you are alone	86.1% N = 67	93.6% N = 73
3.Correct pulse to verify presence in unconscious adult patient	97.7% N = 76	98.7% N = 77
4. Maneuver to perform to verify the presence of respiratory activity in an unconscious patient	78.7% N = 61	96.2% N = 75
5. Actions to do by medical staff in case of absence of vital signs	68% N = 53	93.6% N = 73
6.Correct ration between chest compression and ventilation during CPR in adult patient	50.4% N = 39	98.7% N = 77
7. Correct point on chest to put hand on to perform CPR	23.6% N = 18	94.9% N = 74
8. When to switching on semi-automatic defibrillator and analysis of cardiac rhythm	80.3% N = 62	82.9% N = 64
9. Correct sequence to use semi-automatic defibrillation	32% N = 25	85.1% N = 66
10. Correct position of defibrillator pads on adult chest	56.8% N = 44	92.3% N = 72
11. Shockable rhythm	55.9% N = 43	94.7% N = 74
12.Semiautomatic defibrillation indicates that shock is indicated -Action to do before shock delivery	19.3% N = 15	93.6% N = 73
13.Semiautomatic defibrillation indicate that shock is NOT indicated-action to do	51% N = 38	88.5% N = 69
14.Correct actions to do in unconscious patient with no vital signs until arrival Intensive care specialist	82.9% N = 64	100% N = 78
Average of correct answers	62.3	93.7

## Discussion

In‐hospital cardiac arrest still represents an important health problem associated with significant mortality. Early CPR and defibrillation are the mainstays of cardiac arrest treatment and all hospital personal should know how to correctly perform cardiopulmonary resuscitation. Education and regular training have a crucial role in increasing patient’s survival. Consequently, a further ring was added to the “chain of survival”, leading to the definition of the “chain of prevention” [16]. Education represents the first step of “*chain of prevention”* without which subsequent steps fail with predictable negative effect on patient outcome [16]. Unfortunately, several surveys have already demonstrated the scarce knowledge of medical students regarding CPR and other lifesaving techniques across Europe [17, 18]. Sadly, what has emerged from the current literature is the significant lack of education and skills also in hospital personnel [19–21]. Similarly, our study has evidenced the necessity of adequate training for medical and nurse staff in cardiac arrest management. After training session, operators felt more confident with a positive impact on knowledge. Consequently, national initiatives have to be promoted in order to improve the spread of cardiopulmonary resuscitation protocols. In this perspective, simulation can play a crucial role [22]. A recent guidance note by the European Resuscitation Council, published in European Journal of Anesthesiology, highlighted the importance of teaching CPR technique to healthcare students [23]. However, not only healthcare personnel need to be educated about lifesaving maneuvers. In fact, a key role is played by the community response to out-of-hospital cardiac arrest in order to increase survival rate. In fact, the importance of training the children on CPR was strongly supported by WHO through the “KIDS SAVE LIVES” program; a 2 h-course on CPR from the age of 12 years annually [24].

In our study, we found that participants had difficulties answering correctly to the majority of the questions presented. Before our training, the average of correct answer about the latest guideline was 62.3% with a significative increase after training course (93.7%). An alarming fact that emerged from our study is that the majority of our participants were not able to respond correctly at several fundamental aspects of the BLS-D algorithm. Sadly, only 23.6% were able to respond properly to the question about the correct hands position on chest during CPR. These data indicated a poor familiarity with cardiac arrest management protocol. Furthermore, only half of the interviewed answered correctly about the compressions and ventilations ratio: the others half answered with rates recommended in previous guidelines. We can assume that almost half of the staff involved did not receive any information about CPR guidelines after 2005. This findings are in line with the study of De Almeida et al. [25]. Additionally, it is worth to note that the overwhelming majority of the participants failed to answer correctly about the sequence of steps for operating a defibrillator and what to do in case that a shock is indicated or not. After our training sessions, we appreciated a significant increase in the percentage of correct answers, even if the increased obtained were slightly lower than the other questions. From these data, we can postulate that the use of defibrillation were not rapidly metabolized by all staff involved. Our results were consistent with the study of Lirola et al. [26]: the authors observed that only 31% of the participants were able to use defibrillator properly and only 25% answered correctly about the right treatment of ventricular fibrillation and asystole. During our practical training session, we used both AED trainer and manual defibrillator for demonstration purpose. We decided to use both defibrillators because of the high heterogeneity of the participants enrolled in our survey (e.g., different background, working in different settings). Due to the heterogeneity of equipment available in their clinical settings, we believed that it was vital to explain the different function of both defibrillators*.* All participants were allowed to perform the BLS-D sequence and use the defibrillators on the manikins; each group of participants spent about 1 h of training. Reasonably, the limited duration of the course and the heterogeneity of the participants (i.e., critical and non-critical area) might explain the low confidence at the end of the course on defibrillator usage.

This study has several limitations that should be acknowledged. First of all, the duration of the course was not in line with the official BLS-D course. Even if the duration of BLS-D course was not determined in literature and it seems more important to review protocol periodically in order to reduce skills decay, we believe that the duration of the course could potentially explain why at the end of the course, 51.3% of respondents still considered their preparation not entirely appropriate. As reported by the 2020 International Consensus on Cardiopulmonary Resuscitation Emergency Cardiovascular Care Science with Treatment Recommendations, skills tend to decline over time (i.e., 3–12 months after training) [27]. Consequently, review and updates should be constant in order to improve performance and reduce skill decay. However, our main aims were to raise awareness and, at the same time, to evaluate the general knowledge and skills of health care personnel working in our hospital. An internal survey is essential in order to highlight critical issues and promote solutions. Consequently, we structured the course as "information meetings" and not as a certified course. We also believed that an informal meeting would be attended by a larger part of the staff. Second of all, the high heterogeneity of participants; healthcare personnel worked in critical or non-critical areas. We did not perform subgroup analysis on the base of critical expertise. This aspect may have influenced our results. In fact, it would be expected that healthcare personnel working in intensive care unit or in an emergency room had more experience on lifesaving maneuvers in comparison to nurses and medical staff working in non-critical department. Third, the number of participants was limited. We observed a high drop out and we did not investigate the reason why the participants did not show to the course or completed the questionnaire. Consequently, the evidence obtained is scarce. We believe that BLS-D course should be mandatory for all the hospital personnel, not only for healthcare professionals involved in critical area. Even more, skills and knowledge regarding lifesaving techniques should be refreshed and tested periodically.

## Conclusions

In our study, we found that medical and nursing staff considered their skills on CPR and defibrillation not appropriated. The significant training necessity had also emerged from the analysis of the questionnaires revealing the inadequate knowledge about the latest guidelines. Despite these negative findings, we observed that our informative meeting had had a positive impact on the participants. The percentage of the participants that considered adequate their preparation at the end of the course rose significantly. Even more, healthcare personnel were willing to attend further training course on lifesaving manoeuvres. A certified BLS-D course organized by instructors who are dedicated to CPR training has to be mandatory for every healthcare personnel working in every hospital. Even more, we strongly believe that internal surveys are essential in order to highlight critical issues and promote solutions. Each hospital should take internal survey periodically about crucial issue (such as lifesaving manoeuvres) in order to bring to light problems, find and implement solutions and check results.

## Supplementary Information

**Additional file 1: Material 1**. Questionnaire “A”: Pre course questionnaire regarding personal experience in cardiac arrest management. BLS: Basic Life Support; ALS: Advanced Life Support; ATLS : Advanced Trauma Life Support; OR : Operating Room; ROSC: Return Of Spontaneous Circulation ; ICU: Intensive Care Unit. **Additional file 1: Material 2**. Questionnaire “B”: ERC BLS-D guideline knowledge

## Data Availability

Not applicable.

## Abbreviations

IHCA: In-hospital cardiac arrest
RRT: Rapid Response Team
CPR: Cardiopulmonary resuscitation
ILCOR: International Liaison Committee on Resuscitation
BLS-D: Basic life support and defibrillation
BLS: Basic life support
ERC: European Resuscitation Council
ALS: Advanced life support
OR: Operating room
AED: Automatic external defibrillator
WHO: World Health Organisation
