# Supplementary material for: Assessment of the knowledge level and experience of healthcare personnel concerning CPR and early defibrillation: an internal survey
Source: BMC Cardiovasc Disord. 2021 Apr 20;21:195. doi: 10.1186/s12872-021-02009-2 (PMC8056553; doi:10.1186/s12872-021-02009-2)
Supplement: Supplementary file 1 — Additional file 1: Material 1. Questionnaire “A”: Pre course questionnaire regarding personal experience in cardiac arrest management. BLS: Basic Life Support; ALS: Advanced Life Support; ATLS : Advanced Trauma Life Support; OR : Operating Room; ROSC: Return Of Spontaneous Circulation ; ICU: Intensive Care Unit. Additional file 1: Material 2. Questionnaire “B”: ERC BLS-D guideline knowledge [file 12872_2021_2009_MOESM1_ESM.docx]

**Supplemental Material 1. Questionnaire “A”: Pre course questionnaire regarding personal experience in cardiac arrest management**

| **Question** | **Possible answers** | | | | | | | | |
| --- | --- | --- | --- | --- | --- | --- | --- | --- | --- |
| 1. Qualification | Nurse | | | | Doctor | | | | |
| 2. Years of work | <5years | | 6-10 years | | | | >10 years | | |
| 3. Ever attended a BLS course? | Yes | | | | No | | | | |
| 4. Ever attended ALS course? | Yes | | | | No | | | | |
| 5.Ever attended ATLS course? | Yes | | | | No | | | | |
| 6.Ever attended a company course on in –hospital cardiac arrests management? | Yes | | | | No | | | | |
| 7.Do you have personal experience on cardiac arrest management? | Yes | | | | No | | | | |
| 8.Do you know that in your ward an emergency trolley is available? | Yes | | | | No | | | | |
| 9. Do you know what you can find in an emergency trolley? | Yes | | | | No | | | | |
| 10.Do you know that in your ward a defibrillator is available? | Yes | | | | No | | | | |
| 11. Are you able to use a defibrillator? | Yes | | | | No | | | | |
| 12.Have you ever attended an in-hospital cardiac arrest? | Yes | | | | No | | | | |
| 13. How many in-hospital cardiac arrests have you attended? | *Write the number* | | | | | | | | |
| 14. Where did it happen? | In my ward | | In other ward | | In the OR | | | | In the waiting room |
| 15. What the outcome of the last cardiac arrested that you witness? | ROSC | | | | Die | | | | |
| 16. How did you take part in the cardiac management? | Use of defibrillator | Called the ICU- team | | Called the -ICU team and take part of the management | | | | Not take part of the management | |
| 17. Why did you not take part of the cardiac arrest management? | ICU personnel already present | | | | Did not know what to do | | | | |
| 18. How can you judge your preparation on cardiac arrest management? | Good | | Sufficient | | | Not sufficient | | | |
| 19. Do you consider useful a course on cardiac arrest management? | Useful | | Quite useful | | | Not useful | | | |

BLS: Basic Life Support; ALS: Advanced Life Support; ATLS : Advanced Trauma Life Support; OR : Operating Room; ROSC: Return Of Spontaneous Circulation ; ICU: Intensive Care Unit

**Supplemental Material 2. Questionnaire “B”: ERC BLSD guideline knowledge**

| **Questions** | **Possible answers** | | | |
| --- | --- | --- | --- | --- |
| 1. In patient with cardiac arrest, how long after permanent damage due to anoxia appears in neurons? | Few seconds | 1 minute | 5-10 minutes | 30 minutes |
| 2. During your shift, an adult patient become unconscious. Nobody is available to help you. What is the best thig to do in your opinion? | Verify conscience, breathing and pulse, call the Intensive Care specialist, begin CPR and prepare for defibrillation | Call the Intensive Care specialist and wait for his arrival | After evaluate the absence of vital sign begin one minute of CPR and then call Intensive Care specialist | Verify and resolve upper airway obstruction and then call the Intensive Care specialist and wait for his arrival. |
| 3. In case of unconscious adult patient, what is the correct pulse to verify the presence? | Radial | Carotid | Brachial | Femoral |
| 4. In order to verify the presence of respiratory activity in a unconscious patient , what is the correct maneuver to perform immediately? | Chest auscultation | Put the ear on patient mouth and listen for respiratory sound for 10 seconds | Put the ear on patient mouth and listen for respiratory sound for 10 seconds, at the same time look at patient chest to observe expansion | Look at patient chest to observe expansion for 10 seconds |
| 5.In case of absence of vital signs, medical staff should: | Insert IV access and administer adrenalin 1 mg | Perform 12 lead ECG | Perform CPR and rapidly obtain a defibrillator | Perform CPR |
| 6. Correct ratio between chest compression and ventilation during CPR in adult patient: | 15:2 | 30:2 | 5:2 | 5:1 |
| 7. Correct point on chest to put hand to perform CPR: | On sternum near xiphoid process | On sternum at center of the chest | On sternum slightly on the left | On sternum at clavicles junction |
| 8. Switching on semi-automatic defibrillator and analysis of cardiac rhythm have to be performed in case of: | Unconscious patient that still breathing | Conscious patient with clinical sign of cardiac disease | None of above because semi-automatic defibrillator use is exclusive competence of cardiologist or Intensive Care specialist | Unconscious patient is not breathing and has no signs of circulation |

| **Questions** | **Possible answers** | | | |
| --- | --- | --- | --- | --- |
| 9. The correct sequence to use semi-automatic defibrillation is | Switch on defibrillator-apply pads on chest- connect pads to defibrillator-start analysis-provide shock if necessary | Connect pads to defibrillator-start analysis -apply pads on chest -provide shock if necessary/ | Connect pads on chest- ally pads on defibrillator- switch on defibrillator- start analysis-provide shock if necessary | Don’t remember the sequence |
| 11. Shockable rhythm are: | Atrial fibrillation | Asystole | Ventricular fibrillation and ventricular tachycardia without pulse | Ventricular fibrillation and asystole |
| 12. If semiautomatic defibrillation indicates that shock is indicated, what do we have to do before shock delivery? | Administer adrenaline 1 mg IV | Pay attention that people around patients do not touch the patient | Pay attention that people around patients do not touch the patient and there are no oxygen sources nearby | Pay attention that who is delivery the shock do not touch the patient |
| 13. If semiautomatic defibrillation indicate that shock is NOT indicated, what do we have to do? | Re-check vital signs, if absent start CRP | Re-check pulse, if absent we wait for Intensive Care specialist for death ascertainment | Perform CPR for 2 minutes until defibrillator communicate the necessity to re-analyze rhythm | Re-check breathing activity, if absent perform just ventilations |
| 14. If you are alone with an unconscious patient that is not breathing and without pulse, while waiting for Intensive care specialist, what are you going to do? | Mask ventilation | CPR | Large bore IV access and adrenaline administration | None of above, I will go finding defilbrillator independently by the time take to find it |

CPR: cardiopulmonary resuscitation; IV: intravenous
